# Conserved Mechanisms in the Formation of the Airways and Alveoli of the Lung

**DOI:** 10.3389/fcell.2021.662059

**Published:** 2021-06-15

**Authors:** David Warburton

**Affiliations:** The Saban Research Institute, Children’s Hospital Los Angeles, University of Southern California, Los Angeles, CA, United States

**Keywords:** airway, alveolus, branching, morphogenesis, conserved

## Abstract

Branching is an intrinsic property of respiratory epithelium that can be induced and modified by signals emerging from the mesenchyme. However, during stereotypic branching morphogenesis of the airway, the relatively thick upper respiratory epithelium extrudes through a mesenchymal orifice to form a new branch, whereas during alveologenesis the relatively thin lower respiratory epithelium extrudes to form sacs or bubbles. Thus, both branching morphogenesis of the upper airway and alveolarization in the lower airway seem to rely on the same fundamental physical process: epithelial extrusion through an orifice. Here I propose that it is the orientation and relative stiffness of the orifice boundary that determines the stereotypy of upper airway branching as well as the orientation of individual alveolar components of the gas exchange surface. The previously accepted dogma of the process of alveologenesis, largely based on 2D microscopy, is that alveoli arise by erection of finger-like interalveolar septae to form septal clefts that subdivide pre-existing saccules, a process for which the contractile properties of specialized alveolar myofibroblasts are necessary. Here I suggest that airway tip splitting and stereotypical side domain branching are actually conserved processes, but modified somewhat by evolution to achieve both airway tip splitting and side branching of the upper airway epithelium, as well as alveologenesis. Viewed in 3D it is clear that alveolar “septal tips” are in fact ring or purse string structures containing elastin and collagen that only appear as finger like projections in cross section. Therefore, I propose that airway branch orifices as well as alveolar mouth rings serve to delineate and stabilize the budding of both airway and alveolar epithelium, from the tips and sides of upper airways as well as from the sides and tips of alveolar ducts. Certainly, in the case of alveoli arising laterally and with radial symmetry from the sides of alveolar ducts, the mouth of each alveolus remains within the plane of the side of the ductal lumen. This suggests that the thin epithelium lining these lateral alveolar duct buds may extrude or “pop out” from the duct lumen through rings rather like soap or gum bubbles, whereas the thicker upper airway epithelium extrudes through a ring like toothpaste from a tube to form a new branch.

## Introduction

The vertebrate airway arises during early embryonic organ development from the laryngotracheal groove, a structure comprised of a condensation of epithelial progenitor cells which lies on the ventral surface of the primitive foregut. This airway progenitor population then elongates posteriorly to form the trachea, which separates from the esophagus by a process of lateral septation and epithelial distal to proximal closure to form two distinct tubes, the esophagus and the trachea. Failure of these processes results in a spectrum of rare congenital anomalies including complete atresia of the airway or esophagus, trachea-esophageal fistula, cleft larynx, tracheomalacia or tracheal stenosis (Warburton et al., 2000; Warburton, 2017).

**FIGURE 1:**
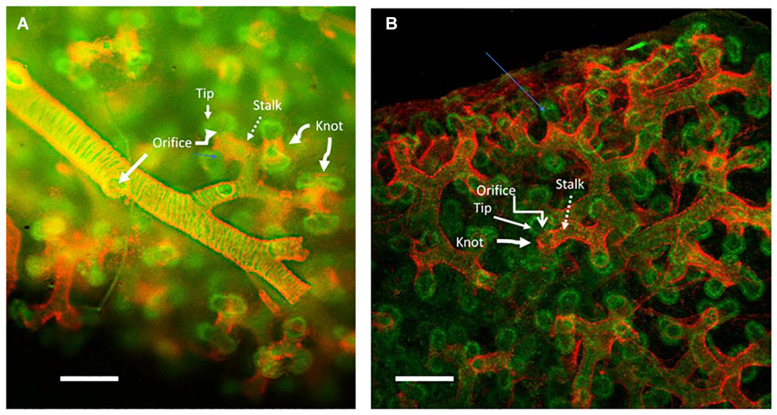
Human lung aged 12 weeks gestation: whole mount specimen stained red for smooth muscle actin marking smooth muscle and green for e-cadherin marking the epithelium and imaged by confocal microscopy. **(A)** The smooth muscle actin (red) sleeve surrounding the peripheral stereotypically branching peripheral main airway epithelium (green) extending from left to tight across the panel. Round holes in the sleeve can be clearly seen where lateral branches arise (marked as Orifice). The sleeve of smooth muscle (marked as Stalk) extends outward toward the distal tips of the epithelium, whereat pairs of tips (marked as Tip) can be seen to extrude through round holes (Orifice) in the smooth muscle sleeve to make new peripheral epithelial branches. Smooth muscle knot-like structures between and around the peripheral tip branches are marked as Knot. **(B)** A more peripheral, juxta pleural view of the more peripheral branching process in the same specimen. The more proximal smooth muscle sleeve (Stalk), the peripheral holes in the sleeve (Orifice) where new branches. Also, the knot-like structure (marked as Knot) of smooth muscle that appears to divide and orient the specific planar direction of epithelial branch clefting and hence of tip extrusion. The plane of the pleura is at the top of the image. The images are adapted with kind permission from Danopoulos et al. (2020). Scale bars 100 microns.

The left and right mainstem bronchi arise from the distal end of the primitive trachea. Subsequent rounds of distal tip branching as well as side branching from the airway establish a stereotypical pattern of left or right lobar and segmental branches. Distal to these stereotypical branches the alveolar ducts branch again several times before ending up at the pleural surface. Branching morphogenesis of the proximal airway in human fetal lung is illustrated in Figure 1.

**FIGURE 2:**
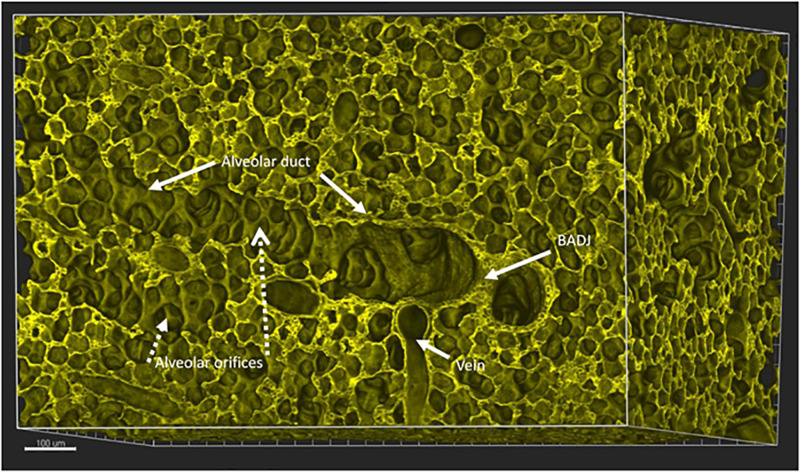
Mouse lung aged 14 days postnatal, a time by which alveolarization has substantially progressed in mice. A digital 3D reconstruction of a whole mouse lung imaged by Vibra-SSIM technology. Membrane fluorescence is imparted by the tomato transgene. This stereo-optical view shows a broncho-alveolar duct junction (marked as BADJ), viewed en face and identified by the sharp columnar to flattened epithelial transition. Two alveolar ducts are viewed proceeding distally to this BADJ structure, one posteriorly and interiorly while a second duct (marked as Alveolar duct) proceeds toward the left from the BADJ. Many individual alveolar orifices (marked as Alveolar orifices) are seen arising from the lateral surface of this alveolar duct. In this 3-dimensional view the mouth or orifice of each alveolus or air sac is surrounded by a round purse string like structure that circumscribes the entrance to each alveolus. Some of the purse strings can also be viewed in oblique cross section, where they appear as the tips of finger-like structures, previously delineated in the literature as “alveolar septae.” A pulmonary vein is also viewed in 3D. Alveolar capillaries can also be discerned in cross section between alveolar surfaces. Scale bar 100 microns.

During the phase of alveolarization, the air sacs form both at the tip as well as the majority forming from the sides of these distal alveolar ducts. The orientation of alveoli along alveolar ducts distal to the bronchoalveolar duct junction (BADJ) in mouse lung is illustrated in Figure 2.

In this short perspective I discuss the fundamental molecular processes of stereotypical airway morphogenesis: progenitor cell induction, tube lengthening and widening, tip branching and side branching, while drawing parallels with the process of alveologenesis formed similarly from the tips and sides of apparently randomly space-filling branches of the distal ducts.

The developmental processes of airway and alveolar formation require the correct sequential function of key transcriptional factors as well as coordinated signaling by growth factors between mesenchyme and epithelium (Warburton et al., 2000; Morrisey and Hogan, 2010; Ardini-Poleske et al., 2017; Warburton, 2017). These must be coordinated with properties of the adjacent capillary vasculature and the extracellular matrix. Together these factors co-opt certain fundamental and quite simple mathematical rules of physics to make a stereotypically branched tubular structure, distal to which non-stereotypic space filling branching and alveolar budding make a large and efficient interface to facilitate gas exchange between the airway and the pulmonary capillary bed (Guo et al., 2014; Bokka et al., 2015a,b; George et al., 2015).

Induction of respiratory progenitors is likely the earliest prerequisite for lung morphogenesis. Both retinoic acid receptor and Nkx2.1 (TTF1) transcriptional activation are absolutely required for the epithelial progenitors of the laryngotracheal groove and proximal bronchi to form correctly (Minoo et al., 1995; Warburton et al., 2000; Warburton, 2017). The first tip splitting event is the formation of the left and right mainstem bronchi at the carina. In mice loss of function of the FGF signaling ligand FGF10 or of its cognate receptor FGFR2b abolishes bronchial branching just distal to the carina (Bellusci et al., 1997; Min et al., 1998; Sekine et al., 1999; Warburton et al., 2003). Whereas in human loss of FGF10 or loss of function of FGF signaling produces a milder syndrome termed Lachrymo-auricular-digital dysplasia (LADD).

The timing and stereotypical orientation of subsequent induction of lobar and segmental airway branches are determined by mesenchymal inductive as well as repressive cues. Before E15 in mouse, supernumerary branches and indeed lobes can be induced from the side of the trachea by ectopic transplantation of distal lung mesenchyme (Shannon et al., 1998). Moreover, apparently directionally random and exuberant branching of isolated mouse embryonic lung epithelial organoids can be induced by FGF10 added to the culture medium (Bellusci et al., 1997). Meanwhile, directional migration of early embryonic lung epithelial organoids can be induced to move chemotactically toward and can even engulf a nearby placed FGF10 bead (Bellusci et al., 1997).

Thus, while branching *per se* appears to be an intrinsic property of the respiratory epithelium, both induction and repression of branching are key processes in determining the stereotypic pattern of branching. Lateral primary branching of the airway stem has been termed Domain branching, whereas distal tip splitting has been termed planar or orthogonal depending upon the plane of orientation of the split (Metzger et al., 2008; Warburton, 2008).

The direction of stereotypical tip branching is determined in large part by the extracellular matrix, in particular the orientation of fibronectin, integrins, and Wnt signaling (De Langhe et al., 2005). Branch tips extend outwards from their origin until a branching “clock” strikes. Then the tip stops extending, while two or occasionally three branch buds form in the stereotypic proximal airways (Unbekandt et al., 2008). These buds then begin to move laterally to the direction of expansion, then turn the corner to move distally to form mostly two, but sometimes three new branches. Meanwhile the interbranch cleft recedes proximally away from the daughter tips as a result of tip outgrowth and smooth muscle and/or, myofibroblast contractility. Later in development, distal alveolar ducts can form as many as five new branches dividing from the same branch tip, especially at the BADJ.

Airway tube lengthening and widening are two further distinct but complementary molecular processes that both require cell proliferation but likely in different planes of cellular cleavage. In the stems the cellular plane of cleavage is lateral, which drives expansion of the branch lumen (Warburton et al., 2010; Tang et al., 2011).

The scaling of interbranch lengths and the number of branches is tailored to the eventual size of the lung across species. In mice the interbranch length is quite short and the number of branch generations is relatively smaller, while in humans these dimensions are much longer and branch generations are correspondingly more numerous. Control of interbranch lengths and timing of branch points depend at least in part on the oscillation of calcium waves, which have been proposed to function as cellular clocks (Jesudason et al., 2010). Calcium waves that mediate peristaltic waves of smooth muscle contraction are conducted from proximal pacemakers in the upper airway down the airway smooth muscle coat and mediate rhythmic contractions that pull proximally on the inter-tip mesenchyme, displacing the interbranch cleft proximally by several cell diameters. Similar calcium waves are also transmitted laterally between cells within the epithelium in response to mechanical forces. How many times these putative branching clocks strike and thus the eventual size of the branch tree may be in turn determined by interaction with such tissue sizing genes as Hippo, YAP, and TAZ (Isago et al., 2020; Warburton, 2020).

Collections of airway progenitor cells are situated at extending airway tips as well as appearing as local condensations at the sites of lateral domain buds. Tip planar and lateral domain bud progenitors contain concentrations of signaling molecules that can both positively and negatively modulate pathways leading to cell cycle activation vs. arrest as well as cell migration vs. stasis. These are known to include several dyadic signaling networks such as FGF receptors, Shc, Grb, SOS, Myc, and Sprouty2 as well as other pathways including EGF receptors, TGF beta and BMP receptors as well as their respective downstream Smad effector and inhibitor signaling mechanisms such as Gremlin plus transcription factors such as Sox2, Sox9, and Id2 (Miettinen et al., 1997; Tefft et al., 1999, 2002, 2005; Mailleux et al., 2001; Chen et al., 2005; Danopoulos et al., 2018).

Vascular hemato-endothelial progenitor cells that express VEGF receptors are also found in intimate association within both the tip clefts as well as at side domain branching locations of the airway and may play an important inductive and mechanically supportive role. Inhibition of VEGF signaling with a dominant negative VEGFR construct inhibits branching, in particular the postero-anterior domain branches (Del Moral et al., 2006; Lazarus et al., 2011).

In human embryonic lung, smooth muscle cells form a distinct sleeve that surrounds the outside of extending branches (Danopoulos et al., 2018). Lateral branching does not occur where the smooth muscle sleeve is present. At each branching tip and lateral domain bud the smooth muscle cells form a ring or orifice that appears to be stabilizing and playing a key role in dividing the tip branch into two or sometimes three, as well as in orienting the lateral domain bud so that each new tip can extrude outwards directionally, like toothpaste out of the mouth of a tube, into the surrounding mesenchyme to form new, stereotypically orientated branches (Figure 1). Moreover, the positional presence of the smooth muscle sleeve suggests that it may suppress the condensation of progenitors to form any new lateral buds where they are not supposed to be.

The functional association between smooth muscle and bud formation is particularly striking in early human lung as shown in Figure 1 (Danopoulos et al., 2018). In early human fetal lung, smooth muscle forms a sleeve around the airway. At points of lateral budding there is an orifice in the smooth muscle coat through which the lateral bud epithelium protrudes and appears to extrude. Meanwhile at branch tips smooth muscle appears to form a knot like structure within the branch cleft so that the epithelium appears to extrude laterally through a pair of laterally placed orifices, rather in the manner of tying a bow tie around one’s neck (Danopoulos et al., 2018). Under video microscopy, these knot structures are pulled proximally by waves of smooth muscle contraction.

However, in mice with genetic ablation of myocardin, smooth muscle differentiation appears to be dispensable for branching to proceed, while mesenchymal differentiation of the tracheal cartilage and neurogenesis are strongly disrupted (Young et al., 2020). This suggests that redundant mechanisms such as myofibroblast contractility can potentially compensate for the absence of smooth muscle contraction and peristalsis in setting branch patterns (Li et al., 2020). Moreover, in mice distal smooth muscle sleeves and knots are not as striking as in human. Since in mouse smooth muscle differentiation and contraction is not essential and may not even be particularly important, I suggest that in the absence of smooth muscle differentiation, the matrix and mesenchyme surrounding branch points must supply the necessary mechanical and molecular signals and constraints to control local mesenchymal stiffness and epithelial adherence in a way that facilitates branching. However, in the genetically intact mouse, as in larger animals such as humans and sheep, smooth muscle expression and rhythmic calcium driven contraction waves do seem to be extremely important for airway peristalsis and overall lung growth.

Whilst the lung is developing *in utero*, rhythmic peristaltic contractions of airway smooth muscle move hydraulic waves of fluid distally down the tube lumen of the developing lung. These waves arrive at the tips and distend them. Blockade of the calcium oscillations that drive these contraction waves arrests branching and this is reversible when the calcium blockade is lifted, and this branching control mechanism is conserved all the way back to fruit fly tracheal branching morphogenesis (Bower et al., 2017). In addition, the extracellular calcium receptor CaR plays an important role in regulating both mouse and human lung development and appears to work by association with the CFTR ion channel (Brennan et al., 2013, 2016). Moreover local parasympathetic innervation is important in controlling branching, in a conserved mechanism from flies to mice that depends on innervation rather than actual neurotransmission (Bower et al., 2014).

In mice there is a precise point of epithelial transition between the upper stereotypic airways and the distal space filling alveolar ducts, termed the BADJ. Albeit, in humans the transition is less precise, and both the bronchial cartilage and airway progenitor cells extends relatively more deeply into the lung, as does the more distal extent of distribution of Club and basal cells. At the BADJ in mice there is a precise transition between the cuboidal and relatively thick upper airway epithelium and the much thinner peripheral alveolar duct epithelium, and then a sharp transition to the even thinner gas exchange epithelium at the alveolar mouth. Thus, effacement or thinning and flattening of the epithelium from relatively thick in major airways to relatively thin and flattened in the alveoli may be an important mechanism in determining the transition from airway branching to alveolar duct branching to alveolar formation.

**FIGURE 3:**
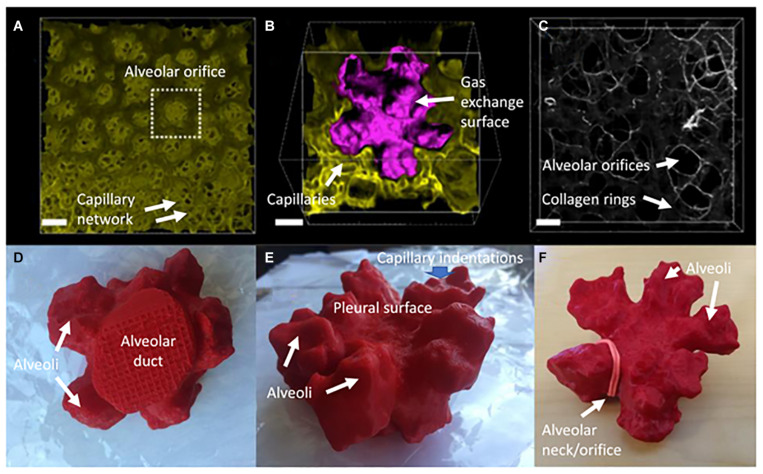
Some key characteristics of peripheral alveoli of the mouse lung at 14 days postnatal age. **(A)** Digital reconstruction of a Vibratome serial section imaging microscopy (Vibra-SSIM) image of peripheral air sacs and their subjacent alveolar capillary plexi, looking outward from the mouth of the air sacs toward the pleural surface. The mouse in question has green membrane fluorescence. Scale bar is 50 microns. **(B)** The family of air sacs surrounded by the dotted box in **(A)** is shown enlarged with its gas exchange surface digitally rendered in purple to give contrast with the green background image. At this magnification the lumena of alveolar capillaries can be clearly seen in cross section within the lung mesenchyme. There are 5 alveoli connected to a common central lumen and this is typical for alveolar structures adjacent to the pleura. The irregular shape and surface rugosity of individual alveoli are also clearly shown. Scale bar is 25 microns. **(C)** Shows second harmonic generation (SHG) in white of collagen fibers located within the mesenchyme of alveolar mouths. Several groups of 3 or 5 air sacs are delineated by interconnected SHG collagen purse strings surrounding the mouths of individual air sacs. In some of them a bed spring like structure can be seen to extend proximally up the neck of a group of air sacs, surrounding the most distal portion of the alveolar duct. Scale bar is 25 microns. **(D–F)** Show 3-dimensional printed views of these air sacs rendered in purple in **(B)** and shown here as complementary 7 oblique views of a red plastic 3-dimensional solid object. **(D)** Shows the proximal to distal view of the round alveolar duct leading into a family of 5 alveoli. **(E)** Shows an oblique view of the irregular and rugose surface of the distal pleural surface of the family of 5 alveoli. **(F)** Shows a distal to proximal view of the five interconnected air sacs. An elastic band has been placed around the mouth of one air sac to illustrate where the collagen mouth rings shown in **(C)** lie in relation to the alveolar orifice.

**FIGURE 4:**
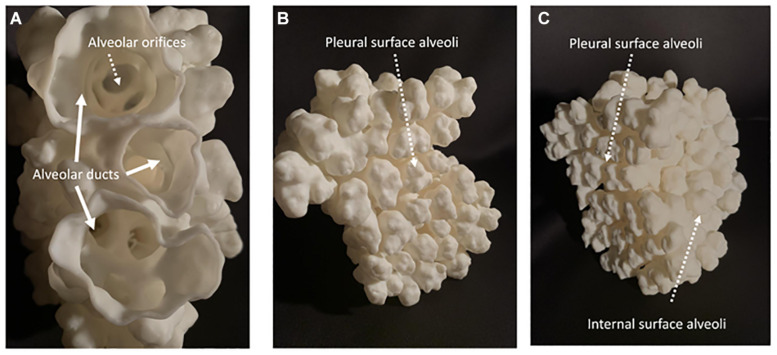
Shows a 3D printed construct of the surface of alveoli and distal alveolar ducts in a 28 days postnatal age distal mouse lung acinus. **(A)** Shows a proximal to distal view down three branches of the same distal alveolar duct looking down through their alveolar orifices into families of distal alveoli. **(B)** Shows the topography of distal alveolar families adjacent to the pleural surface. **(C)** Shows an oblique view of the 3D construct with both the pleural surface and the adjacent interior surface alveoli clearly visible.

New alveoli arise from the alveolar ducts in two distinct patterns: distal and lateral. The distal alveoli arise from the tip of each duct as a Fibonacci rosette usually comprising five alveoli, interconnected at their base by interlocking rings of elastin and collagen. Meanwhile, the lateral alveoli that actually form the majority of the gas diffusion surface, bud out from the sides of the alveolar ducts, rather like brussels sprouts budding with radial symmetry from their parent stalk (Figures 1–4).

The widely accepted dogma of the process of alveologenesis, largely based on extrapolation from 2D microscopy, is that alveoli arise by erection of finger-like interalveolar septae that form septal clefts that subdivide pre-existing saccules, a process for which the contractile properties of specialized alveolar myofibroblasts are necessary (Burri, 2006; Schittny, 2017). Meanwhile the correct deposition and cross linking of elastin fibers in the tips of these septa is required, all under the control of locally and correctly modulated PDGF ligand-receptor signaling. In mice either deficient or super abundant PDGF signaling severely disrupts alveolar myofibroblast function, elastin deposition and thus leads to abnormally hypoplastic alveologenesis (Boström et al., 2002).

In human as in murine early lung development, stereotypical branching morphogenesis is completed *in utero* while the lung contains non-compressible hydraulic fluids. Alveologenesis also begins *in utero* in humans under fluid filled positive pressure conditions, whereas in mice it is mainly a postnatal process that proceeds under the intermittent negative pressure conditions of air breathing. In both species the vast majority of alveoli form postnatally between babyhood and adulthood in the air breathing lung, from the sides of alveolar ducts. Moreover, the size and surface area of alveoli remains remarkably stable over the course of postnatal lung development in both mice and humans, implying that while early alveolarization may indeed occur by subdivision of pre-existing saccules, another process must be invoked to explain the stability of alveolar dimensions in the postnatal lung.

Here I would like to suggest an alternative or perhaps complementary hypothesis that the process of airway tip splitting and of stereotypical side domain branching are actually conserved processes, but modified somewhat by evolution to achieve both airway tip splitting and side branching as well as alveologenesis. Viewed in 3D (Figures 1–4) it is clear that alveolar “septae” are in fact ring or purse string structures that only appear as finger like projections in cross section (Barré et al., 2016; Branchfield et al., 2016; Surate Solaligue et al., 2017; Schittny, 2018). Therefore, I speculate that these alveolar mouth rings serve to delineate and stabilize the budding of alveoli both from the tip as well as from the sides of alveolar ducts. Certainly, in the case of alveoli arising laterally and with radial symmetry from the sides of alveolar ducts, the mouth of each alveolus remains within the plane of the side of the ductal lumen. This suggests that the thin epithelium lining these lateral alveolar duct buds may extrude or “pop out” from the duct lumen rather like soap or gum bubbles (Figures 1–4). Clearly the surface tension lowering properties of pulmonary surfactant would be conducive to facilitating this process as well as for stabilizing the alveolar epithelium and preventing collapse once alveoli have budded out.

Experimental support for this idea comes from more recent 3D visualization over time (4D) of *de novo* alveologenesis in precision cut lung slices in culture (Akram et al., 2019) and Sucre, personal communication). In this system new alveoli begin as a condensation of epithelial cells that migrate into foci at the intersections between adjacent alveoli. These condensations then begin to hollow out to form a nascent, annular bird’s nest shaped structure, which then expands and thins to bud outwards to form a new alveolus. The walls of these new alveolar buds do indeed grow in height, but outwards from the duct lumen and appear to be stabilized at their mouth by a purse string-like ring of elastin and latterly collagen fibers (Luo et al., 2018). Failure of these alveolar purse string rings to form causes alveolarization to fail under such conditions as gain or loss of function of PDGF receptor signaling or in response to increased perinatal oxygen exposure.

Pathologically, hypo-alveolarization is associated with failure of correct alveolar purse string formation and this is a cardinal feature of bronchopulmonary dysplasia in human premature infants as well as in cases of pulmonary hypoplasia secondary to congenital diaphragmatic hernia, as well as being found in certain congenital diseases of the lung elastic matrix such as Marfan’s and Loitz-Dietz syndromes. Meanwhile, destruction of alveolar mouth rings is a cardinal feature of apparently adult onset emphysema.

Thus, I propose herein that both branching morphogenesis of stereotypic proximal airways as well as the emergence from alveolar ducts of tip and stalk alveoli, seem to form by a conserved mechanism of extrusion of the epithelium through a directionally orientated orifice with a ring or purse string-ring lip, that imparts some localized stiffness to the mesenchyme. Thus, the alveolar epithelium extrudes outwards into the surrounding mesenchyme, which is correspondingly less stiff than the alveolar orifice. I further propose that the lung matrix and the local capillary network contribute both inductive cues as well as some matrix and hydraulic structural geodesic stiffness properties which support the directional expansion of the epithelium to form new tubes in both stereotypic as well as more distal non-stereotypical branching. Also, in the case of alveolarization similar mechanisms contribute to epithelial expansion to form and stabilize a thin and efficient alveolar gas diffusion surface, which in turn is maintained by the surface tension lowering properties of pulmonary surfactant.

## Data Availability Statement

Publicly available datasets were analyzed in this study. This data can be found here: LungMAP.

## Ethics Statement

The studies involving human participants were reviewed and approved by IRB Children’s Hospital Los Angeles. The patients/participants provided their written informed consent to participate in this study. The animal study was reviewed and approved by IACUC Children’s Hospital Los Angeles.

## Author Contributions

DW conceived and wrote this article.

## Conflict of Interest

DW serve on advisory boards regarding pediatric interstitial lung disease at Boehringer Ingelheim.
